# Thickness of the buccal bone wall and root angulation in the maxilla and mandible: an approach to cone beam computed tomography

**DOI:** 10.1186/s12903-018-0652-x

**Published:** 2018-11-21

**Authors:** P. López-Jarana, C. M. Díaz-Castro, A. Falcão, C. Falcão, J. V. Ríos-Santos, M. Herrero-Climent

**Affiliations:** 1 Porto Dental Institute, Porto, Portugal; 2 Marbella Dental, Marbella, Spain; 3 School of Dentistry. Universidad de Sevilla (Spain), C/Avicena S/N, 41009 Seville, Spain

**Keywords:** Buccal bone wall thickness, Basal bone angulation, Root angulation, Basal bone, Cone beam computed tomography, CBCT

## Abstract

**Background:**

The objective of this paper is to anatomically describe the bone morphology in the maxillary and mandibular tooth areas, which might help in planning post-extraction implants.

**Methods:**

CBCT images (Planmeca ProMax 3D) of 403 teeth (208 upper teeth and 195 lower teeth) were obtained from 49 patients referred to the Dental School of Seville from January to December 2014. The thickness of the facial wall was measured at the crest, point A, 4 mm below, point B, and at the apex, point C. The second parameter was the angle formed between the dental axis and the axis of the basal bone.

**Results:**

A total of 403 teeth were measured. In the maxilla, 89.4% of incisors, 93.94% of canines, 78% of premolars and 70.5% of molars had a buccal bone wall thickness less than the ideal 2 mm. In the mandible, 73.5% of incisors, 49% of canines, 64% of premolars and 53% of molars had < 1 mm buccal bone thickness as measured at point B. The mean angulation in the maxilla was 11.67 ± 6.37° for incisors, 16.88 ± 7.93° for canines, 13.93 ± 8.6° for premolars, and 9.89 ± 4.8° for molars. In the mandible, the mean values were 10.63 ± 8.76° for incisors, 10.98 ± 7.36° for canines, 10.54 ± 5.82° for premolars and 16.19 ± 11.22° for molars.

**Conclusions:**

The high incidence of a buccal wall thickness of less than 2 mm in over 80% of the assessed sites indicates the need for additional regeneration procedures, and several locations may also require custom abutments to solve the angulation problems for screw-retained crowns.

## Background

According to a prospective study, the majority of bone remodelling occurs after a dental extraction in the buccal plate, with a vertical loss of 1 mm and a larger degree of horizontal resorption (80–63%) than vertical (69–65%) [1]. In addition, the mid-buccal recession of an immediate implant placed into a fresh extraction socket has been reported to be 0.55 to 0.75 mm at 1 year of follow-up [2].

The immediate (after tooth extraction) implant placement protocol [3] has advantages over other early or delayed approaches in the reduction of treatment time and patient discomfort, since it requires fewer surgical procedures. The assessment of the tooth root anatomy helps the clinician to properly choose the best treatment protocol [4]. The thickness of the buccal alveolar bone wall, especially the bundle bone (whose vascularization depends on the periodontal ligament [5, 6]), undergoes extensive remodelling during the healing of the alveolar process. This influences soft and hard tissue volume and its relationship with the implant, which impact the biological and aesthetic results achieved [7].

The planning of an immediate implant requires an intact vestibular wall or a type 1 socket as rated by Elian et al. [8], which means a socket where the vestibular and palatal plates and the overlaying soft tissue are preserved. Furthermore, at least a 2-mm thickness of the vestibular plate is needed for soft tissue stability to prevent prosthetic and aesthetic complications. Many authors suggested that the shoulder of the implant should be placed in the area that they called the comfort zone: 1.5 or 2 mm apical to the gingival margin of the future restoration [9]. A more buccal position carries a greater risk of recession and restorative difficulties [10]. Placing it too palatal results in an overlapping or over-contouring towards the vestibule. The morphology of the residual alveolar process is also crucial to determine the orientation for implant placement [11]. Carvalho described the bone triangle concept, which consists of the residual basal bone apical to the alveolar process [12]. The implant position could be affected by the angulation of this basal bone triangle, which in turn is vital to achieve primary stability for an immediate implant. The difference between the proper emergence of the implant crown and the ideal angulation of this apical bone triangle should be 10 degrees [13].

Many clinical situations require additional surgical procedures, apart from all the previously mentioned considerations, to make screw-retained prostheses. Apical fenestrations of the bone plate in the anterior maxillary region are common when leading the emergency screwed profile into the palatal zone [14]. Previous anatomic studies have shown deep depressions in the alveolar bone around the apex, which becomes a risky situation, especially in the lateral incisor region due to the limited availability of alveolar bone [15]. However, some clinicians prefer to preserve the thickness of the alveolar process by positioning the implant in the same tooth axis and afterwards restore them with CAD-CAM or standard angulated abutments [16].

CBCT helps to establish the morphologic characteristics of the residual alveolar process [17–19].

The main aim of this study is to anatomically describe the bone morphology in the maxillary and mandibular alveolar bone tooth areas, which might help in planning post-extraction implants. The analysis consists of assessing [1] roots position of remaining teeth in the alveolar process by measuring the distance from the root to the buccal wall at three specific locations, and [2] the angle formed by the axis of the basal bone with the axis of the tooth.

## Methods

The present transversal descriptive study included CBCT images obtained by an x-ray device (Planmeca ProMax 3D; Planmeca Oy; Helsinki, Finland) using a spiral technique with 0.2 mm thickness (200 μm voxel size, 200 mm field of view (FOV), 90 kV, 10 mAs, 1 mm pass) from patients referred to the Periodontology Department of Dental School of Seville for implant therapy from January to December 2014. The ethical committee for the University of Seville approved this non-interventional study for the acquisition of the images, number 0159-N-14 (PEIBA) of the Junta de Andalucía, Spain. The inclusion criteria are described in Table 1.

**Table 1:** The systemic inclusion and local exclusion criteria

Systemic inclusion criteria	Local exclusion criteria
1. Absence of systemic disease of relevant history of bad health (particularly ruling out bone diseases, uncontrolled or poorly controlled diabetes, unstable or life-threatening conditions or requiring antibiotic prophylaxis).	1. Radiolucent image greater than 1/3 of the root, metal artefacts.
2. Absence of history of radiotherapy or chemotherapy in the past 5 years	2. Severe root angulation (selected tooth image was not contained in slice).
3. Absence of autoimmune diseases and any drug use.	3. Severe root resorption.
4. Absence of pregnancy or lactation.	4. Radiographic evidence of surgical (guided bone/tissue regeneration) treatment in the anterior maxillary dentition.

### Image process and codification

The CBCT images used in the present study were selected from the faculty’s database and were not specifically acquired for this study. The CBCT images were anonymous and were saved in Digital Imaging and Communications in Medicine (DICOM) format inside a protected computer with specific software for implant planning. The measurements were performed using a commercial image analysis and graphics software (Adobe Photoshop CS5, Adobe Systems Incorporated, 345 Park Avenue, San Jose, California 95, 110, USA) by 3 pre-calibrated surgeons. The captured images of the scan were saved with the standard zoom and resolution of Planmeca Romexis viewer of Planmeca ProMax 3D; and exported to Adobe Photoshop CS5, to be measured.

### Examiner calibration

Three examiners were calibrated using 10 randomized CBCT images on 2 different days, 48 h apart. The calibration was achieved by blind measurements of the same random teeth by the three examiners, registering the grade of reproducibility. The intra-examiner intraclass correlation coefficient (ICC) were 0.98, 0.97 and 0.98, and the inter-examiner ICC were 0.99 and 0.98.

### Radiographic image analysis of the CBCTs

CBCT images were analysed on two computers, both with Windows 7 and Intel core i-7 processors with a monitor resolution of 1366 × 768. Data were reconstructed with an image size of 401 × 401 × 401, voxel size 200 μm, 90 Kv, 14 mA, 12,249 s and DAP 12,3 (mGyxcm^2^).

**Fig. 1:**
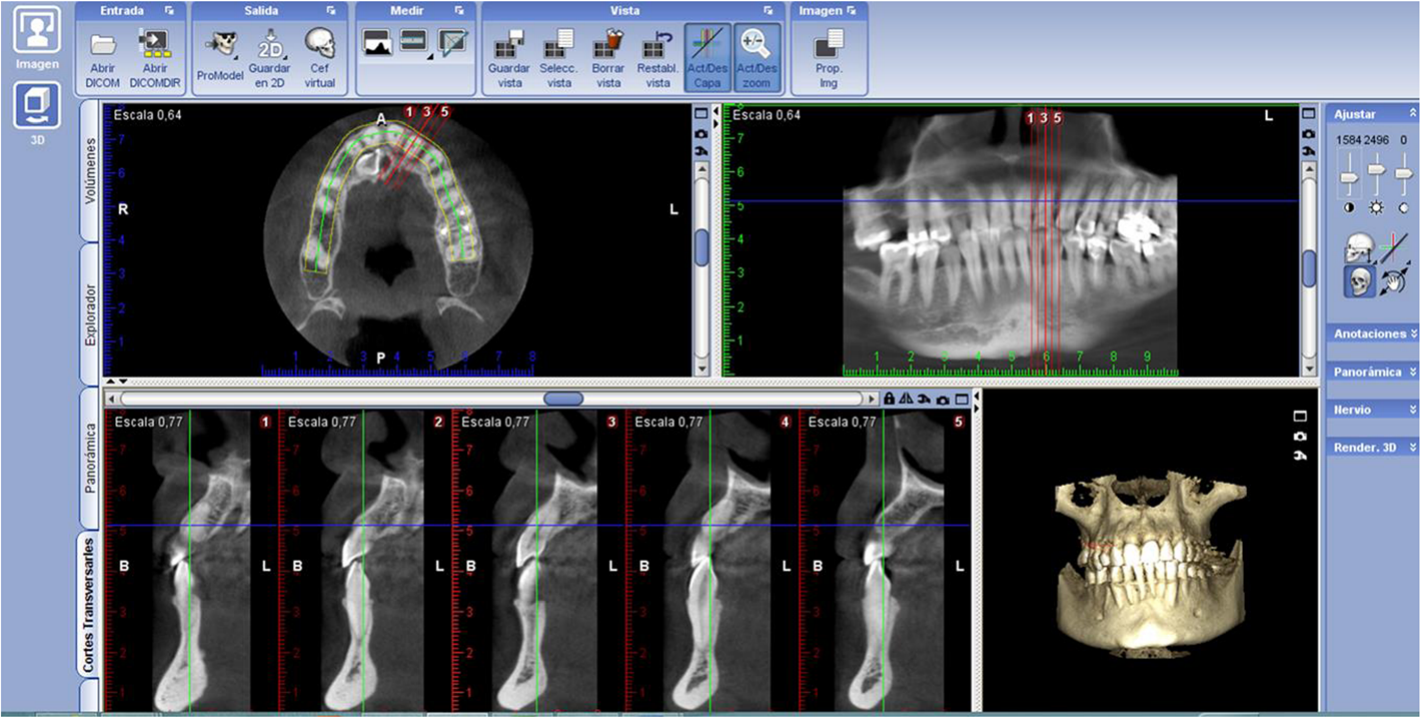
Selection of the axial slice

**Fig. 2:**
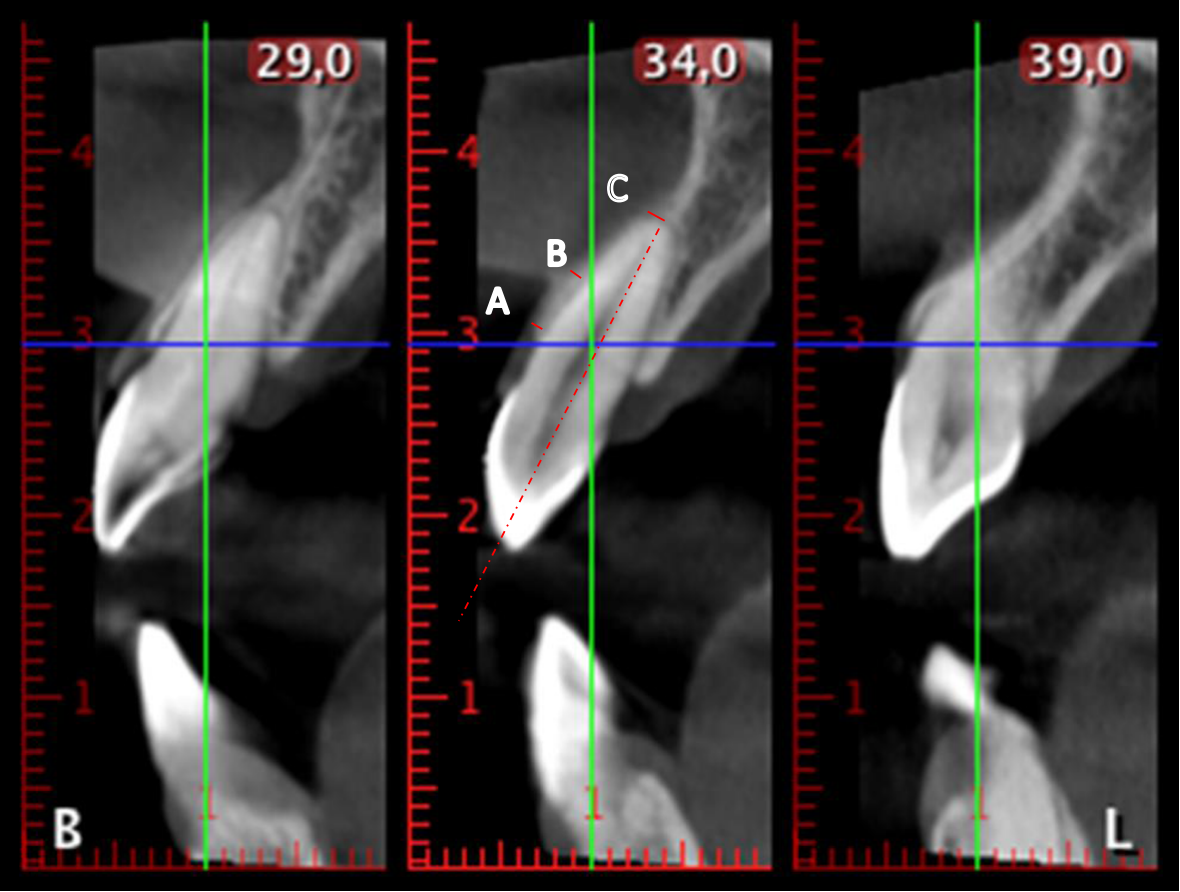
The thickness of the buccal bone wall. **a** Thickness at the coronal part of the buccal crest. **b** Thickness at 4 mm from the coronal buccal crest. **c** Thickness at the apex from the apical constriction to the buccal wall

The arch form selector tool was centred throughout the middle of the arch in the coronal plane at the cement-enamel level selecting the centre of the nerve canal of single root teeth and the middle of the interradicular septum of multirooted teeth (Fig. 1). The thickness of the alveolar bone was measured after selecting the cross-sectional image made at the midpoint of the tooth, at which the centre of the root canal passes, parallel to its long axis (Fig. 2). To perform the measurements, sagittal scans from the reconstructed data were selected, resulting in images where the entire root and cementoenamel junction (CEJ) were present for single rooted teeth. Two different slices were selected for multirooted teeth, one which passed across from the apex of the mesiobuccal and the distobuccal root. The long axis determined the slice. The captured images had a resolution of 72 pixels/inch and were saved with the standard zoom of Planmeca Romexis [20] viewer and exported to Photoshop CS5 to be measured. All the images had a lateral ruler which served to the surgeons for calibrating the measurements made on the photo editor to the distance at the DICOM images.

### Parameters or variables

**Fig. 3:**
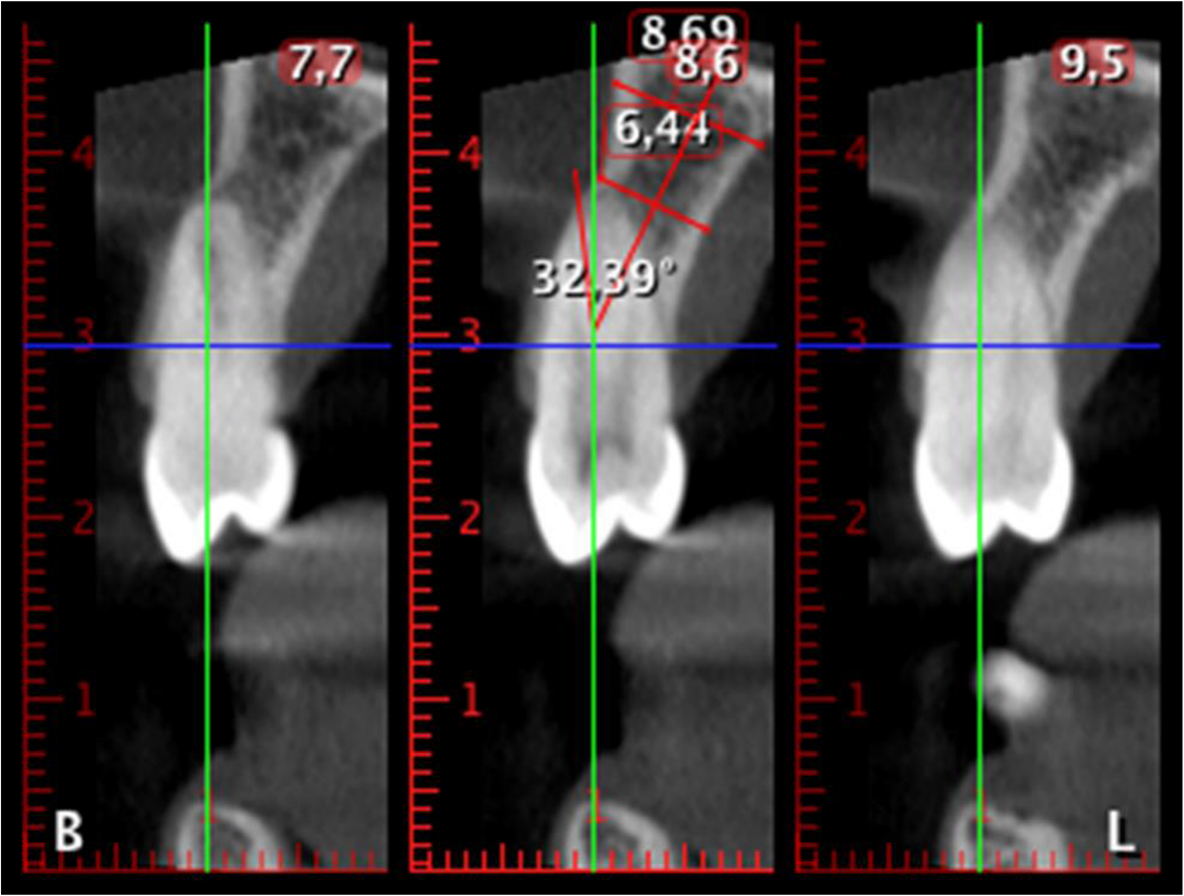
The angle between the dental axis and the basal bone

The thickness of the buccal bone wall was measured in a sagittal slice, perpendicular to the long axis of the root (defined by the line from the incisal border to the apex) at several points (Fig. 2):

A: Thickness at the first top coronal part of the buccal crest,
B: Thickness 4 mm upper from the point A.
C: Thickness at the apex from the apical constriction to the buccal wall.
  Measurement of the angle formed by the axis of the basal bone triangle with the axis of the tooth in the upper and lower arches (Fig. 3).
  The angle of the basal bone triangle was defined by two reference points in the palatal and vestibular areas at the apex and other two points in the apical part of the basal bone triangle. A perpendicular line passes across these horizontal lines at the middle. With the angle tool, the axis of the tooth and the axis of the basal bone were drawn, and the angle formed in their intersection was determined.
  The authors have uploaded the Excel data file to the ‘idUS’ repository of the University of Seville. Available for readers.

### Statistical analysis

The data obtained was introduced in Excel software (Microsoft) to perform a descriptive analysis with the adequate codification of the patients. The data were analysed using SPSS software version 22. Descriptive statistics, including the mean, SD, and 95% confidence intervals (CIs), were calculated.

## Results

A total of 49 patients (mean age of 40.3 years) met the inclusion criteria (19 men and 30 women), resulting in a sample of 403 teeth that were measured. Of these, 208 were upper teeth and 195 were lower teeth. In the maxilla, the samples included 32 central incisors, 34 lateral incisors, 33 canines, 25 first and 25 s premolars, and 31 first and 28 s molars. In the mandible, the samples included 39 central incisors, 38 lateral incisors, 35 canines, 37 first and 25 s premolars, and 10 first and 11 s molars.

### The thickness of the buccal bone

In the maxilla, the buccal alveolar plate of premolars and molars was the thickest, measured at each reference point. Approximately 89.4% of incisors, 93.94% of canines, 78% of premolars and 70.5% of molars had a thickness lower than the ideal 2 mm of the buccal alveolar process (Table 2).

**Table 2:** Medium values of the thickness of the alveolar process

Table 2	μ	A	B	C
Maxillary incisors	66	1.036 ± 0.46 mm	1.021 ± 0.49 mm	1.614 ± 0.95 mm
Maxillary canines	33	1.047 ± 0.39 mm	1.27 ± 1.95 mm	1.26 ± 0.68 mm
Maxillary premolars	50	1.20 ± 0.67 mm	1.43 ± 0.95 mm	2.19 ± 1.68 mm
Maxillary molars	59	1.240 ± 0.83 mm	1.55 ± 1.41 mm	2.153 ± 1.68 mm
Mandibular molars	21	0.917 ± 0.416 mm	3.109 ± 2.03 mm	6.779 ± 2.925 mm
Mandibular premolars	62	0.841 ± 0.402 mm	1.490 ± 0.97 mm	3.814 ± 1.827 mm
Mandibular canines	35	0.794 ± 0.407 mm	1.079 ± 0.86 mm	3.535 ± 1.869 mm
Mandibular incisors	77	0.767 ± 0.361 mm	0.936 ± 0.77 mm	3.187 ± 1.905 mm

In the mandible, the buccal alveolar plate of premolars and molars was also the thickest, measured at each reference point. A mean thickness of the buccal bone of < 1 mm was present in 57 incisors, 24 canines, 40 premolars and 11 M in site A, but this thickness increased to > 2 mm in 54 incisors, 26 canines, 54 premolars and 12 M in site C. At site A, 74.02% of the incisors, 68.57% of canines, 64.51% of premolars and 57.14% of molars were < 1 mm at the first measurement points. To understand the distribution of the bone thickness at the wall, the study sample was divided into two groups: ideal (≥2 mm) and non-ideal thickness (< 1 mm) (Table 3).

**Table 3:** Distribution of the buccal bone wall thickness in groups

Dental group	<1MM			1–2 MM			> 2 mm		
	A	B	C	A	B	C	A	B	C
Max Incisors (C, L)	35 (53–53%)	32 (53–44%)	15 (13–32%)	26 (38–41%)	31 (44–50%)	34 (59–44%)	5 (9–6%)	3 (3–6%)	17 (28–24%)
Max Canines	18 (45%)	19 (58%)	12 (36%)	12 (30%)	13 (39%)	17 (52%)	3 (8%)	1 (3%)	4 (12%)
Max Pm (1°, 2°)	20 (54–24%)	18 (56–16%)	12 (36–12%)	27 (42–64%)	21 (36–48%)	15 (28–32%)	4 (4–12%)	10 (8–32%)	25 (36–56%)
Max M (1°, 2°)	25 (45–32%)	28 (55–39%)	19 (48–14%)	25 (45–39%)	13 (23–21%)	15 (26–25%)	11 (10–29%)	18 (23–39%)	25 (26–61%)
Mnd Incisors (central, lateral)	57 (79–68%)	47 (62–61%)	5 (5–8%)	20 (21–32%)	26 (33–34%)	18 (31–16%)	0	4 (5–5%)	54 (64–76%)
Mnd Canines	17 (49%)	15 (43%)	3 (9%)	2 (6%)	16 (46%)	6 (17%)	16 (46%)	4 (11%)	26 (74%)
Mnd Pm (1°PM, 2°PM)	40 (68–60%)	23 (46–24%)	2 (5–0%)	22 (32–40%)	23 (35–40%)	4 (5–8%)	0	16 (19–36%)	54 (84–92%)
Mnd Molars (1°M, 2°M)	11 (70–36%)	0	0	10 (30–64%)	9 (80–9%)	0	0	12 (20–91%)	21 (100–100%)

### The angulation between the axis of the teeth and the alveolar process

**Fig. 4:**
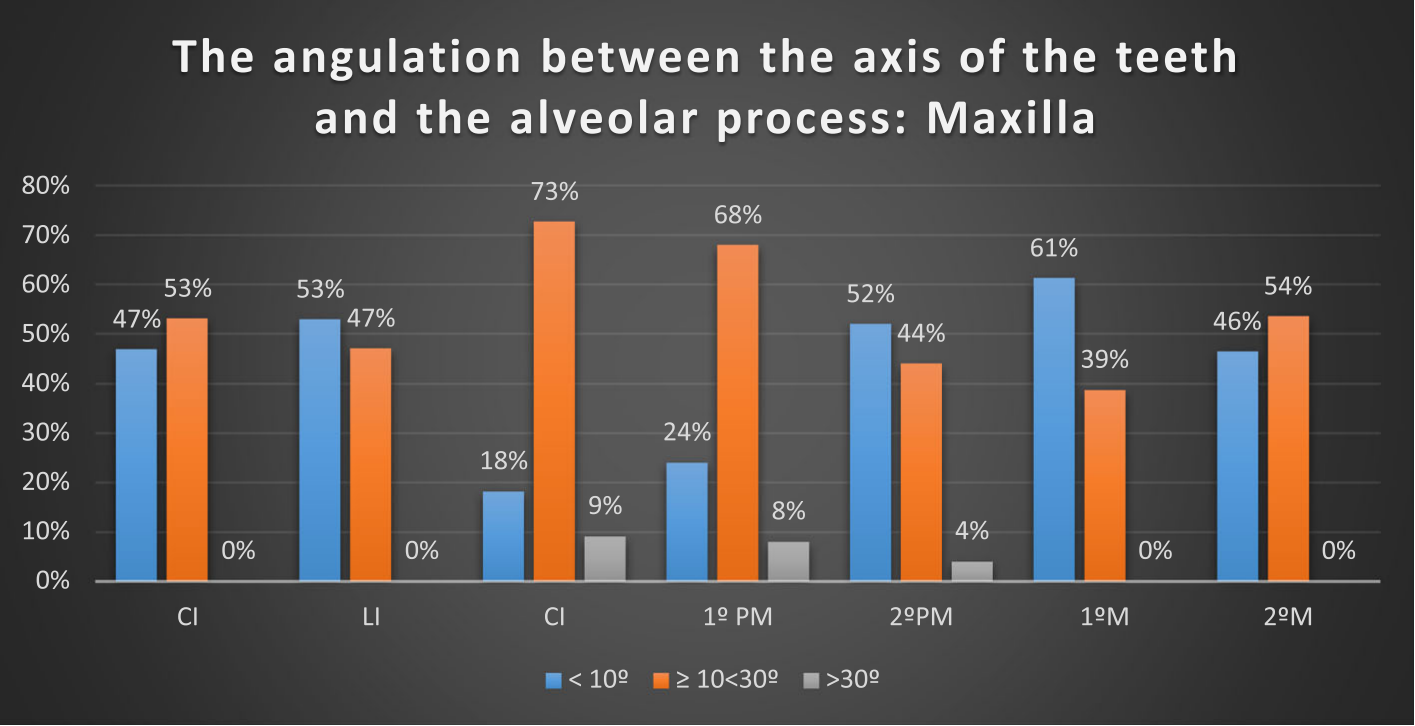
Angulation of the alveolar process versus the axial superior tooth axis

**Fig. 5:**
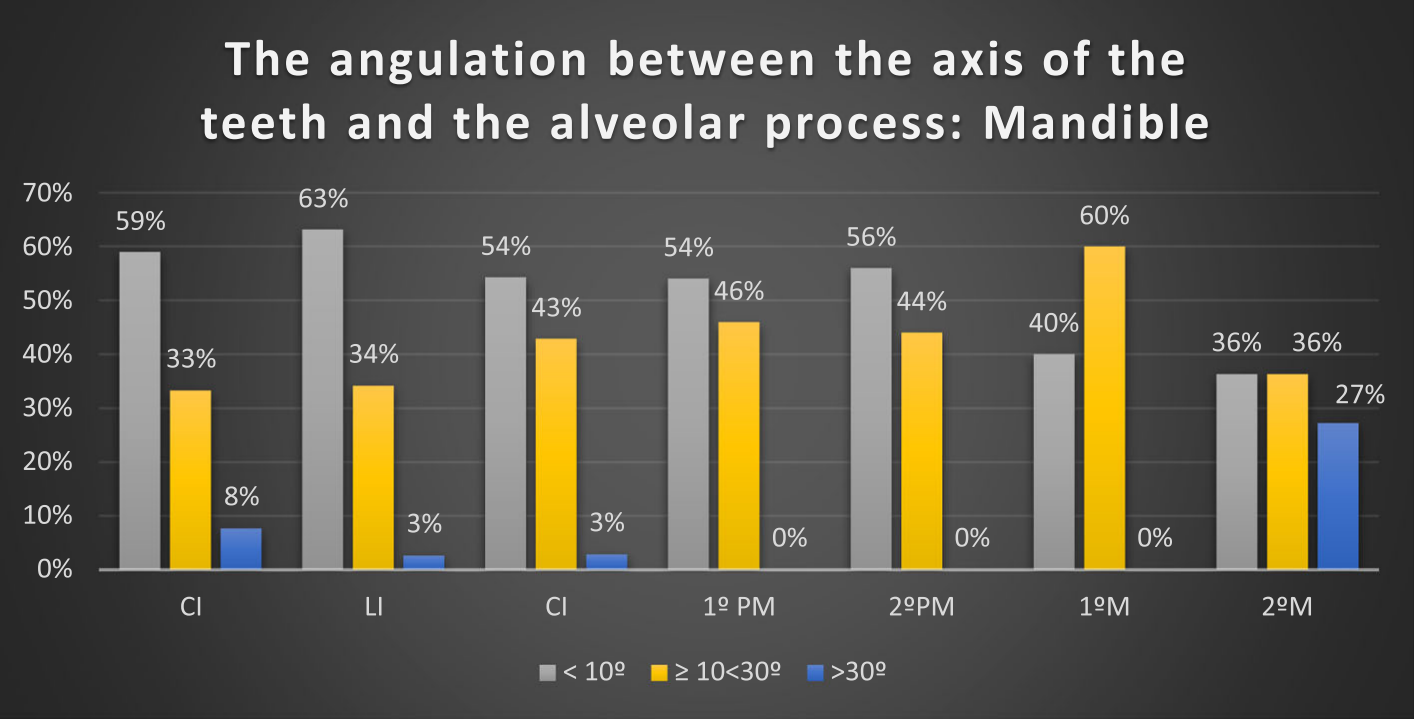
Angulation of the alveolar process versus the axial inferior tooth axis

In the maxilla, only 31 of 66 incisors, 6 of 32 canines, 18 of 47 premolars and 32 of 58 M had an angle less than 10°. In the mandible, the maximum angulation was found in some molars (43.26°) and incisors (38°) (Table 4, Figs. 4, 5).

**Table 4:** Angulation of the alveolar process versus axial teeth axis: group distribution

Table 4	μ	Median	Min	Max
Maxillary Incisors	66	11.67 ± 6.4°	0.70°	27.01°
Maxillary Canines	33	16.88 ± 7.9°	2.18°	34.20°
Maxillary Premolars	50	13.93 ± 8.6°	0.33°	42.60°
Maxillary Molars	58	9.89° ± 4.8°	2.13°	20.90°
Mandibular Incisors	77	10.64 ± 8.8°	1.06°	38.00°
Mandibular Canines	35	10.99 ± 7.4°	2.00°	32.78°
Mandibular Premolars	62	10.54 ± 5.9°	2.05°	25.35°
Mandibular Molars	21	16.19 ± 11.2°	2.81°	43.26°

## Discussion

The long-term aesthetic implications of bone remodelling after implant placement are focused in the apical migration of the vestibular gingival margin, a parameter associated with the disappearance of the bundle bone. Therefore, knowledge of the bone dimensions surrounding the tooth are required in order to predict the degree of reabsorption that will occur after tooth extraction and replacement [17]. The underlying surrounding bone morphology of an immediate implant plays a critical role in soft tissue stability and influences the aesthetic outcome with the final restoration [18]. Many factors are responsible for the possible aesthetic risk when immediate implants are placed: the absence of the bundle bone, the remodelling of the alveolar process after tooth extraction, the thickness of the vestibular bone wall, as well as the convexity of the alveolar process because these two parameters influence the emergence profile of the restoration [21].

The related disparity of the CBCT between the true value and the general mean value was 0.8–1% for width measurements and 2.2% for height measurements [22]. In our study, the CBCT software used did not allow the measurement of a thicknesses less than 0.8 mm; therefore, we had to use Adobe Photoshop CS5 software to measure lower thickness values, which were then converted to real measures.

In the maxilla, the anatomical descriptive study from Huyn-Va et al. in 2010 [19] employed a sample of 93 extraction sockets to show a wall thickness of less than 1 mm in 87% of cases at a coronal level in the portion closest to the cement-enamel junction (CEJ). The current study presents similar results; the thickness of the buccal bone increases from apical to coronal and from the midline to the canine [23].

Our mean results in terms of the thickness of the maxillary buccal bone were as follows: 54.45% of incisors and canines, 60% of premolars and 47% of molars were lower than 1 mm. All values remained between 0.5 and 1.5 mm with exception of 4 lateral incisors, where the measured thickness was greater than 1.5 mm [24].

Januario et al. 2011 [25] measured the thickness of the buccal alveolar bone at 1, 3 and 5 mm apically from the crest in 250 subjects. The values showed that the thickness was always < 1 mm, and in 50% of the cases, it was inferior to 0,5 mm. In our sample, we found that 53% of incisors presented a thickness of the buccal wall bone less than 1 mm.

In the prospective study from Hassan-Assaf et al., the buccal wall thickness measured preoperatively at 2 mm from the crest resulted in median values that were lower than ours (1.20 ± 0.67 mm): 1,03 mm for canines and premolars [26].

On the other hand, our results were slightly higher than those of recently published works, where the average values for the upper arch teeth were less than 1 mm for all sites, and we did not find statistically significant differences between left and right teeth [15, 27–29].

In upper canines and second premolars, we found a thinner buccal bone wall, which seemed to be similar to the results of other studies. For the upper canines, 58 and 56% of first premolars showed a thickness thinner than 1 mm. Our results are in agreement with those of Rojo et al. [30]. They show that the thickness of the first premolars seems to be less than that of second premolars, although our mean values were lower. The reason for these lower values could be explained by our measurement method with Photoshop CS5.

However, our mean values increased in an apical direction. This may be due to the possible skeletal class differences in the population groups in the study [31].

For the upper molars, our mean values were similar to those of other recent publications. Our results showed that 55% of upper first molars and 40% of second molars had thicknesses thinner than 1 mm [32]. In a specific study of posterior teeth from the maxilla and mandible, the authors show an increasing buccal plate thickness from anterior to posterior and from coronal to apical that was greater in maxillary than in mandibular teeth [33, 34].

As posterior teeth in the mandible and maxilla are sites where immediate implants are also placed, the present study included the assessment of these teeth in addition to anterior teeth.

In the mandible, outcomes were similar except for the more apical points (at 8 mm from the CEJ), which were lower than our values at the apex level. This could be related to the reference measurement because our midpoint was situated 4 mm to the crest.

In relation to the angle of the root axis and basal bone axis, our research found that in the upper jaw, the mean values were as follows: 11.67 ± 6.37° for incisors, 16.88 ± 7.93° for canines, 13.93 ± 8.6° for premolars, and 9.89° ± 4.8° for molars. In the mandible, the mean values were 10.63 ± 8.76° for incisors, 10.98 ± 7.36° for canines, 10.54 ± 5.82° for premolars and 16.19 ± 11.22° for molars. However, a maximum of 43.26° was found in some molars and 38° in some incisors.

According to Nishihara et al., the mean value of the angle of maxillary first premolars was 25.5° and 18.1° for second premolars. The insertion angle of the dental implant may leave a depression in the buccal bone that could induce implant protrusion to the vestibular bone in longer implants [35].

According to Kan et al. (2011), the class I type, in which the root is closer to vestibular wall, represents 81.1% of the cases. These angulations are in most cases greater than 10° from canine to canine. The sagittal position of the tooth in the alveolar bone is important for the clinician to make decisions for implant-based therapy [11].

In 2014, Wang found differences greater than 10° in all groups, even up to 42° in some premolars and upper canines. These differences in the results of angulation were greater than 20° in 50% of anterior teeth (and 40% of the canines > 30°). This could be a consequence of the patient’s skeletal class [36]. In this study, the sagittal angle formed between the long axis of the tooth and the long axis of the alveolar bone was < 10° degrees at only 10% of the teeth in the maxillary aesthetic zone.

In more recent studies, the angulation of the tooth axis is related to the horizontal plane and the buccal bone wall. The more obtuse the angle, the thinner the buccal wall. This situation implies increased risk of perforation of the buccal wall in immediate implant placement [31]. In our opinion, the clinical relevance of these data is that in cases where immediate implants are placed according to bone availability, angulated screwed abutments may be required. In some cases, such as premolars and canines, this might require CAD/CAM-customized components, since available standard angulated screwed abutments are not able to compensate for the resulting discrepancy between angulations. Nevertheless, in the mandible, 8% of incisors and 27% of second molars seem to have angulations bigger than 30° between the basal bone and dental alveolus.

In Lau et al’s study, as mentioned previously, the proportion of incisors positioned more buccal (type B) was 78.8%. Furthermore, 19.4 and 1.8% were positioned midway (type M) and more palatal (type P), respectively. This result was in concordance with Kan’s study [37]. The position of the teeth must be considered because the anatomy of the dental alveolus increases the difficulty of the ideal positioning of the immediate implant. Sometimes, type 1 Kan’s classification means that the teeth are too close to the buccal plate, which is thinner, although palatal bone is preserved and is a suitable zone to achieve bone anchorage.

To our knowledge, no studies have analysed the angulation of mandibular teeth. The mean angulation values between the axis of the teeth and the alveolar process for the mandible were 10.63 ± 8.76° in incisors, 10.98 ± 7.36° in canines, 10.54 ± 5.82° in premolars and 16.19 ± 11.22° in molars. However, a maximum of 43.26° was found in 3 M and 38° in 30 incisors.

Once the analysis of the studied parameters was completed, we identified the most unfavourable situations for immediate implants: the lateral incisors, canines and premolars in the maxilla and the incisors, canines and second molars in the mandible.

## Conclusions

There seems to be a link between the angulation of the root and the alveolar axis, which was greater than 10° in almost all the sites studied.

The thickness of the buccal plate was less than 2 mm in over 80% of the teeth studied.

In the maxilla, the most critical areas were the lateral incisors, canines and first premolars, where the thickness of the buccal wall was less than 2 mm and their angulation with the alveolar process varied between 10 and 30°.

In the mandible, the critical sites were central incisors, lateral incisors, and canines, where the thickness of the buccal wall was less than 2 mm. Furthermore, 27% of the second molars made an angle with the alveolus of > 30°, but the buccal bone wall thickness was in 91% of the measured areas. The results of the study could imply that over 80% of assessed sites could require additional regeneration procedures to preserve hard and soft tissue volumes. Also custom abutments might be necessary to solve the angulation of screw-retained crowns.

## Abbreviations

CAD-CAM: Computer-Aided Design/Computer-Aided Manufacturing
CBCT: Cone Beam Computed Tomography
CEJ: Cementoenamel junction
CIs: Confidence intervals
DICOM: Digital Imaging and Communications in Medicine
FOV: Field of view
ICC: Intraclass Correlation Coefficient
PEIBA: Portal de Ética de la Investigación Biomédica de Andalucía (Spain)
